# Mussel-Inspired Sonochemical Nanocomposite Coating on Catheters for Prevention of Urinary Infections

**DOI:** 10.1021/acsami.4c05713

**Published:** 2024-06-25

**Authors:** Antonio Puertas-Segura, Kristina Ivanova, Aleksandra Ivanova, Ivan Ivanov, Katerina Todorova, Petar Dimitrov, Gianluca Ciardelli, Tzanko Tzanov

**Affiliations:** † Grup de Biotecnologia Molecular i Industrial, Department of Chemical Engineering, Universitat Politècnica de Catalunya, Rambla Sant Nebridi 22, Terrassa 08222, Spain; ‡ Institute of Experimental Morphology, Pathology and Anthropology with Museum, Bulgarian Academy of Sciences, Geo Milev, Sofia 1113, Bulgaria; § Department of Mechanical and Aerospace Engineering, Politecnico di Torino, Corso Duca degli Abruzzi 24, Torino 10129, Italy

**Keywords:** catheter, nanoparticles, gelatin, sonochemistry, antibacterial, antibiofilm

## Abstract

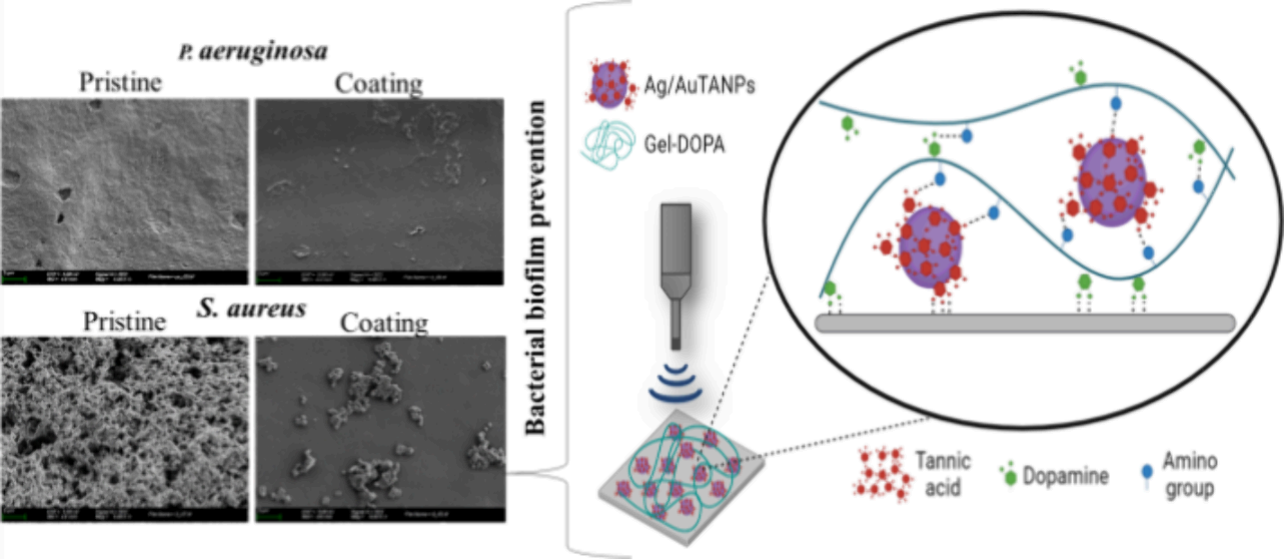

Catheter-associated urinary tract infections are the most common hospital-acquired infections and cause patient discomfort, increased morbidity, and prolonged stays, altogether posing a huge burden on healthcare services. Colonization occurs upon insertion, or later by ascending microbes from the rich periurethral flora, and is therefore virtually unavoidable by medical procedures. Importantly, the dwell time is a significant risk factor for bacteriuria because it gives biofilms time to develop and mature. This is why we engineer antibacterial and antibiofilm coating through ultrasound- and nanoparticle-assisted self-assembly on silicone surfaces and validate it thoroughly in vitro and in vivo. To this end, we combine bimetallic silver/gold nanoparticles, which exercise both biocidal and structural roles, with dopamine-modified gelatin in a facile and substrate-independent sonochemical coating process. The latter mussel-inspired bioadhesive potentiates the activity and durability of the coating while attenuating the intrinsic toxicity of silver. As a result, our approach effectively reduces biofilm formation in a hydrodynamic model of the human bladder and prevents bacteriuria in catheterized rabbits during a week of placement, outperforming conventional silicone catheters. These results substantiate the practical use of nanoparticle–biopolymer composites in combination with ultrasound for the antimicrobial functionalization of indwelling medical devices.

## 1 Introduction

The risk of persistent bacterial colonization increases each day that a urinary catheter is in place and can lead to severe infections and device failure. In fact, catheterization is a major risk factor for complicated urinary tract infections (UTIs), qualifying the latter as a standalone group of catheter-associated UTIs (CAUTIs).^1^ The indwelling devices serve as foreign bodies that support biofilm formation and facilitate the entry of microbes into the bladder. Consequently, frequent catheter replacement and intensive antibiotic therapies are required, which cause patient discomfort and prolonged hospital stays and impose a huge economic burden to the healthcare system.^2,3^ Furthermore, CAUTI organisms are a major source of antimicrobial resistance (AMR) because most catheterized patients receive antibiotics also for other indications, while biofilms set a perfect environment for gene transfer.^4^

The replacement of latex with silicone derivatives, less prone to bacterial attachment, proved inefficient to mitigate CAUTI.^5^ Currently, the most promising material strategies are based on nanostructured coatings^6,7^ of polymers,^8,9^ peptides,^10,11^ enzymes,^12,13^ or metal nanoparticles (NPs).^14−16^ The latter are an important antibiotic alternative due to their diverse mechanisms of action that do not lead to AMR and their strong activity against both Gram-positive and Gram-negative bacteria.^17−19^ Importantly, the blending of metals enables new properties or improves existing ones.^20^ For instance, silver nanoparticles (Ag NPs) exhibit strong antibacterial activity and cytotoxicity, while gold nanoparticles (Au NPs) are considered anti-inflammatory and biocompatible.^21,22^ Their synergy leads to increased stability and larger surface area, promoting the antimicrobial effect, and less toxicity. The synthesis of bimetallic Ag/Au NPs, however, often requires harsh chemicals and reaction conditions that may generate harmful byproducts.^23,24^ A sustainable alternative is the use of natural phenolic compounds as reducing and capping agents, which also provide antioxidant activity and biocompatibility.^25,26^ In parallel, the coating durability should match the dwell time of the catheter. To this end, NPs may be firmly embedded in the device material by local melting or during extrusion, but this strategy is not suitable for thermoset materials such as silicone and may compromise the NP antibacterial mechanism that relies on ion release. One of the most promising approaches for stable but active functionalization relies on ultrasound, which is a fast, economic, and environmentally friendly waterborne process.^27^ The sonochemical coating takes place through cavitation, where the implosion of the generated bubbles projects the NPs toward the surface.^28^ However, limited physical interactions between the substrate and the NPs may still lead to inadequate coating durability.

To mitigate this, we have previously explored different sonochemical strategies, for example, to stabilize ZnO NPs on cotton using enzymes^29^ and bioadhesives like chitosan,^30^ and have translated the latter approach to contact lenses.^31^ We have recently discovered that when embedded in hydrogels made of thiolated hyaluronic acid, lignin-capped Ag NPs not only exercise antibacterial role but also act as dynamic cross-linking nodes.^32^ In the present work, we further exploited the interplay between phenols and NPs, developing a self-assembly approach for antibacterial and antibiofilm coatings on urinary catheters made of polydimethylsiloxane (PDMS). In particular, we first functionalized gelatin with dopamine (abbreviated DOPA) to mimic the molecular mechanism for rapid and strong attachment of mussels to rocks in marine environment.^33^ Since its discovery, this nature-inspired surface chemistry has resulted in multiple coating and adhesive applications because catechol groups ensure wet attachment, largely independent of the substrate.^34,35^ Next, we synthesized bimetallic NPs by reduction with tannic acid (Ag/AuTANPs) and sonochemically deposited them together with gelatin–DOPA, whereby we hypothesized that the phenolic shell of the NPs would enhance the interactions within the composite coating. We used contact angle measurements, Fourier transform infrared spectroscopy (FTIR), and X-ray photoelectron spectroscopy (XPS) to characterize the resulting surfaces as well as inductively coupled plasma (ICP) to quantify the release of Ag. Furthermore, we determined in vitro the antimicrobial activity against *Staphylococcus aureus* and *Pseudomonas aeruginosa* by assessing both planktonic and biofilm-related parameters, including a special setup that mimicked the hydrodynamic conditions of human bladder. Finally, we validated the biocompatibility of the coated catheters in vitro by cytotoxicity studies against model cell lines and in vivo upon a weeklong implantation in rabbits.

## 2 Experimental Section

### 2.1 Materials

Polydimethyl/vinylmethyl siloxane (PDMS, designated as VMQ polymer by ASTM D 1418) urinary catheters and flat sheets of the same material were supplied by Degania Silicone Ltd. Ethanol was purchased from Scharlab. Phosphate buffer saline tablets, SnakeSkin dialysis tubing, *N*-(3-(dimethylamino)propyl)-*N*′-ethylcarbodiimide hydrochloride (EDC), and 2′,7′-dichlorodihydrofluorescein diacetate (DCFH-DA) were provided by Thermo Fisher Scientific. Acros Organics supplied *N*-hydroxysuccinimide (NHS). AlamarBlue cell viability reagent and Live/Dead BacLight kit (Molecular Probes L7012) were obtained from Invitrogen, Life Technologies Corporation. All other chemicals including gelatin, (2-(*N*-morpholino)ethanesulfonic acid hydrate (MES), dopamine hydrochloride, tannic acid, gold(III) chloride trihydrate, silver nitrate, Folin–Ciocalteu phenol reagent, 2,2-diphenyl-1-picrylhydrazyl (DPPH), sodium dodecyl sulfate (SDS), crystal violet, nutrient broth (NB), Muller Hinton Broth (MHB), Tryptic soy broth (TSB), cetrimide agar, Baird-Parker agar, and Dulbecco’s modified Eagle’s medium (DMEM) were purchased from Sigma-Aldrich. Ultrapure Millipore water was used in all of the experiments. Bacteria cultures, i.e., *P. aeruginosa* (ATCC 10145) and *S. aureus* (ATCC 25923) were grown aerobically at 37 °C in TSB.

### 2.2 Functionalization of Gelatin

The conjugation of dopamine hydrochloride to gelatin was carried out by using EDC/NHS. A 1.0 g portion of gelatin was dissolved in 100 mL of degassed MES buffer (pH 4.5, 100 mM) at 37 °C. Then, 575 mg of EDC and 345 mg of NHS were added. After 20 min of stirring, 569 mg of dopamine hydrochloride dissolved in 3 mL of MES buffer (pH 3.3, 100 mM) was added to the mixture, which was then allowed to react at 37 °C in dark with 100 rpm of shaking for 24 h. The resulting solution was purified via a dialysis membrane (MWCO 3500 Da) against acidified deionized water (pH ∼3) four times and finally against deionized water (pH ∼7) for 2 h under vigorous stirring. Then, the functionalized gelatin was lyophilized and stored at −20 °C. The presence of unoxidized catechol groups in the gelatin–DOPA conjugate was assessed using UV–vis spectroscopy.^36^ The phenolic content was determined by the Folin–Ciocalteu reagent as previously described.^37^ Furthermore, gelatin phenolation was also evidenced through free DPPH radical scavenging.^38^

### 2.3 Preparation and Characterization of NPs

Ag/AuTANPs were prepared by reduction, whereby tannic acid was used as a reducing, capping, and stabilizing agent. Initially, a 0.02% (w/v) solution of silver nitrate was prepared in 100 mL of deionized water and heated to 80 °C. Then, 1 mL of 1% tannic acid was slowly added and allowed to stir for 1 h. Following this, 0.02% (w/v) gold(III) chloride trihydrate in 1 mL of deionized water was slowly added to the mixture, and the mixture was stirred for 1 h. After precipitation of the NPs, they were concentrated through centrifugation at 18,000 rpm for 40 min, resuspended in water, and stored at 4 °C. To compare the antimicrobial effect of bimetallic NPs to Au and Ag alone, NPs were generated following variations of the same procedure. The final Ag/AuTANP suspension was characterized by UV–vis spectroscopy (multiwell plate reader, Tecan/Infinite M200). The presence of tannic acid on the NPs was proved by Folin–Ciocalteu phenol reagent, while transmission electron microscopy (FEI Tecnai G2 F20 S-TWIN HR(S)TEM) was used to characterize their morphology. The size distribution from TEM (extracted via image processing in ImageJ) was compared to the polydispersity in aqueous solution from dynamic light scattering (DLS) (Zetasizer Nano Z, Malvern Instruments). The bacterial inhibition was evaluated against *P. aeruginosa* and *S. aureus* following a previously reported protocol.^16^

### 2.4 Coating Characterization

The silicone material was fragmented into 1 × 1 cm pieces and washed sequentially in 0.1% (w/v) SDS, deionized water, and 96% ethanol for 30 min in each step. For sonication, eight silicone samples were immersed in 50 mL of solution composed of 24 mL of Ag/AuTANP suspension and 26 mL of 1% w/v gelatin–DOPA. When individual components were studied separately, the excluded component was replaced by deionized water of the same volume. Ultrasound was applied by a Ti-horn transducer (20 kHz, 750 W, Sonics and Materials VC750) at 20 °C for 30 min with 50% amplitude. After coating, the chemical composition was analyzed by ATR-FTIR (PerkinElmer Spectrum 100 FTIR spectrometer) and XPS. X-ray characterization was performed with a SPECS system, featuring a high-intensity twin anode X-ray source XR50 with Mg/Al (1253/1487 eV) emissions, operating at 150 W. The X-ray source was positioned perpendicular to the analyzer axis, and data acquisition was facilitated by a Phoibos 150 MCD-9 XP detector. The surface morphology was examined via SEM, employing a field-emission scanning electron microscope (Fei Quanta 650 FEG-ESEM) operating at 1 kV and using the low-vacuum mode. Additionally, the elemental composition of the material surface was assessed through energy-dispersive X-ray spectroscopy (EDX). The water contact angle was measured with a DSA 25 Krüss (Germany) via sessile drop and calculated using the tangential method in the Krüss Advanced v1.13.0.21301 software. The same measurement was done after incubation in deionized water at 37 °C with 100 rpm shaking for 7 days. The Ag contents in the Ag/AuTANPs and the coated silicones were quantified using ICP-MS (Model 7800, Agilent). The release profile was evaluated by submerging 1 × 1 cm PDMS samples in 2 mL of sterile artificial urine (produced in accordance with UNE EN1616 for Sterile Urethral Catheters for Single Use) and incubation at 37 °C with 100 rpm shaking. Every 24 h, the silicone pieces were removed and transferred into fresh sterile artificial urine, while the solution containing the released Ag was collected and kept at 4 °C. Ag was then measured by ICP-MS after the addition of 1 mL of 2% nitric acid.

### 2.5 Antibacterial Properties and Mechanism

#### 2.5.1 Antimicrobial Assay for Planktonic Bacteria

The antimicrobial activity of the samples was evaluated using a method adopted from ASTM-E2149-20 with minor modifications.^39^ Single colonies of *P. aeruginosa* and *S. aureus* were grown in 5 mL of sterile MHB at 37 °C and 230 rpm overnight. Cultures were then diluted with a sterile MHB solution to an absorbance of 0.28 ± 0.01 at 600 nm. Subsequently, the solution was diluted 1:1000 in PBS and each 1 × 1 cm sample was incubated with 1.5 mL of the diluted bacterial suspension for 24 h. The resulting colony-forming units (CFUs) were counted by serial dilutions of bacterial suspensions on cetrimide agar and Baird-Parker agar plates for *P. aeruginosa* and *S. aureus*, respectively.

#### 2.5.2 ROS Assay

The ability of the coatings to generate ROS in the solution was measured by a fluorescein derivative DCFH-DA.^40^ To remove the acetate group from the dye, 20 mL of sodium hydroxide (0.01 N) was mixed with 125 μL of DCFH-DA (20 mM) and incubated at room temperature for 30 min in the dark. The process was stopped by adding 100 mL of PBS (25 mM, pH = 7.4). A calibration line was then made by using hydrogen peroxide (10–100 μM). The samples (1 × 1 cm) were incubated in 3 mL of DCFH-DA/NaOH/PBS and then transferred to a 37 °C water bath for 15 min before measuring fluorescence intensity at 490_ex_/520_em_ nm. The same oxidation-sensitive probe was used to measure the production of ROS by bacteria upon contact with the treated silicone. Bacterial cultures of *S. aureus* and *P. aeruginosa* in NB (OD_600_ = 0.5) were exposed to 1 × 1 cm^2^ silicone samples. The mixtures were centrifuged at 4000 g after 4 h at 37 °C and rinsed twice with PBS. The bacteria in the pellet were incubated with 20 mM DCFH-DA solution in PBS for 30 min in the dark, and the fluorescence was measured.

#### 2.5.3 Scanning Electron Microscopy

To study the topographic features of the coatings and their influence on *S. aureus* and *P. aeruginosa* biofilms, 1 × 1 cm samples were incubated with 1.5 mL of bacterial suspension (OD_600_ = 0.01) in TSB in a 24-well microplate for 24 h at 37 °C under static conditions. The biofilm was then fixed by incubating it in 2% paraformaldehyde and 2.5% glutaraldehyde solution overnight. Next, the samples were dehydrated by successive incubations in ethanol with increasing concentrations (25, 50, 75, and 100%) for 10 min each. Due to the nonconductive nature of silicone, the samples were plated with carbon before imaging. Samples without bacteria were not metallized to avoid losing the contrast produced by the particles on the silicone surface.

#### 2.5.4 Quantification of Biofilm Biomass

The inhibition of biofilm development was assessed by the evaluation of the biomass formed by *P. aeruginosa* and *S. aureus* biofilms. Briefly, 1 × 1 cm samples were incubated with 1.5 mL of bacterial suspension (OD_600_ = 0.01) in TSB in a 24-well microplate for 24 h at 37 °C under static conditions, allowing the bacteria to colonize the silicone materials and establish biofilms. After incubation, the resulting samples were washed three times to remove planktonic bacteria with 2 mL of sterile 100 mM PBS (pH 7.4), and then the biofilms were fixed for 2 h at 60 °C. The preserved biofilms were labeled with 1 mL of 0.1% (w/v) crystal violet solution for 15 min. Subsequently, the samples were cleaned with 1 mL of 30% (v/v) acetic acid to dissolve the crystal violet, 125 μL of each sample was transferred to a 96-well microplate, and absorbance was read at 595 nm.

#### 2.5.5 Bacterial Viability in Biofilms

*S. aureus* and *P. aeruginosa* were cultivated in sterile TSB for 24 h at 37 °C to quantify the living cells inside the biofilm. Each material sample was cultured for 24 h at 37 °C in a 24-well sterile plate with 1 mL of bacterial inoculum (OD_600_ = 0.01) in sterile TSB. Following incubation, the samples were washed three times with sterile PBS to remove nonattached bacteria before being transferred into 15 mL sterile tubes containing 2 mL of sterile PBS. The tubes were vortexed for 120 s each, placed in an ultrasonic bath for 20 min, and viable counts were obtained by plating bacterial suspensions on Baird-Parker and Cetrimide agar plates. In addition, samples were treated with the Live/Dead BacLight kit for qualitative assessment. To this end, fluorescence micrographs were recorded at 480_ex_/500_em_ nm for Syto 9 and at 490_ex_/635_em_ nm for propidium iodide.

#### 2.5.6 Hydrodynamic Model of Catheterized Bladder

An in-house-designed experimental setup of a catheterized human bladder was used to evaluate the coating efficiency under realistic hydrodynamic conditions and duration of placement. First, pristine or treated Foley catheters were inserted into the sterile bladder model, and the catheter balloon was inflated with 5 mL of 100 mM PBS, pH 7.4. The bladder was then filled to the catheter eye with sterile artificial urine and supplemented with 1 mg/mL *P. aeruginosa* and *S. aureus* in TSB (OD_600_ = 0.01). The model was maintained at 37 °C for 7 days, and the artificial urine was recirculated at a flow rate of 1 mL/min. Then, the catheter was removed, and the total biofilm mass on the catheter tip or the balloon was measured using crystal violet as described above.

### 2.6 Biocompatibility Assessment

#### 2.6.1 In Vitro Studies

Human foreskin fibroblasts (*BJ-5ta* cell line) and keratinocytes (*HaCaT* cell line) were maintained in four parts of DMEM containing 4 mM l-glutamine, 4500 mg/L glucose, 1500 mg/L sodium bicarbonate, 1 mM sodium pyruvate, and 1 part of Medium 199, supplemented with 10% (v/v) fetal bovine serum, and 10 g/L hygromycin B at 37 °C in a humidified atmosphere with 5% carbon dioxide, according to the recommendations of the manufacturer. The culture medium was replaced every 2 days. At preconfluence, cells were harvested using trypsin-EDTA (ATCC-30-2101, 0.25% (w/v) trypsin/0.53 mM EDTA solution in Hank’s BSS without Ca or Mg) and reseeded. Prior to biocompatibility testing, fibroblasts were plated at a density of 120,000 cells/well in a 24-well tissue culture-treated polystyrene plates. The cells were then placed in direct contact with 1 × 1 cm samples for cytotoxicity assessment. Afterward, 0.75 mL of DMEM was added and the samples were incubated at 37 °C in a humidified atmosphere of 5% carbon dioxide for 1–7 days. At the end of each test, cells were examined for signs of toxicity using an AlamarBlue assay kit, while a blank DMEM sample was subjected to the same conditions as a negative control. To this end, after incubation for 4 h at 37 °C, the absorbance at 570 nm was read by using 600 nm as a reference wavelength. In addition, BJ-5ta viability and morphology were studied by a Live/Dead Viability/Cytotoxicity Assay Kit for mammalian cells after exposure of the cells to the samples for 24 h and 7 days. This kit contains two fluorescent dyes, calcein and ethidium homodimer-1, which allow for simultaneous identification of live (green) and dead cells (red fluorescence).

#### 2.6.2 In Vivo Studies

New Zealand male rabbits (4–5 months old, weighing 3–4 kg) were used to validate the preventive antibacterial/antibiofilm activity of the coating. The animals were hosted in individual cages with free access to food and water. All experimental procedures were carried out in accordance with the national regulation on laboratory animals and animal welfare (No. 20/01.11.2012), the 2010/63/EU directive of the European Parliament, and approved by the Ethical Committee of the Institute of Experimental Morphology, Pathology, and Anthropology with Museum (No. 282/24.09.2020). After 2 weeks of quarantine, the rabbits were medically examined and divided in two groups; the control group 1 (*n* = 3) was catheterized with pristine silicone Foley catheters (French size 8), while the experimental group 2 (*n* = 3) was catheterized with treated Foley catheters (French size 8). The dwell time for both groups was 7 days. The implantation procedure was performed under general anesthesia using a mixture of tiletamine/zolazepam, xylazine, and butorphanol in doses of 5, 4, and 0.15 mg/kg body weight, respectively. Prior to insertion, the treated and untreated catheters were sterilized under UV light for 15 min. Afterward, the catheterization was carried out aseptically. The procedure was performed under ultrasound guidance (Mindray DP-20 Vet). After insertion in the bladder, the outer end of the catheter was cut at 0.2–0.3 cm inside from the urethral orifice, and the catheters were secured with several dermal sutures to the preputial skin. During surgery, standard disinfection was carried out, and the rabbits were left with cervical collars to protect the newly introduced devices. All animals fully recovered after catheterization and were medically examined on each day of indwelling. Blood from the jugular vein was taken before and at the end of the trial, and sterile urine was collected through the catheters and directly from the bladder, right after the euthanasia. Urine was subjected to microbiological tests and urinalysis. Complete blood count and biochemical analysis were performed by an automatic hematology analyzer (Mindray BC 2800 Vet) and blood chemistry analyzer (MNCHIP Celercare V2). Fresh sterile samples of urine (5 mL) from both groups were collected twice as explained above in sterile urine containers. Probes were subjected to a urine analyzer with urine test strips (Urit 50 Vet). Urine sediment smears were done and stained by the May Grunwald Gimsa-rapid method (Diapath, Italy). The materials were examined by a Leica DM 5000B. Fresh sterile urine samples (10 mL) from the beginning and the end of the trial and the cuttings from the middle part of the used catheters were collected aseptically. These were subjected to microbiological analysis in the medical diagnostic laboratory “RAMUS” in Sofia (Bulgaria). After catheterization, all six rabbits were humanely euthanized, and materials for histology were collected from the urethra, bladder, and kidneys. Tissue samples were fixed in 10% neutral buffered formalin, dehydrated, cleared in xylene, and embedded in paraffin. Tissue sections (3–5 μm thick) were stained with hematoxylin/eosin and examined under light microscope. Tissue samples were assessed morphologically for lesions or histopathological signs of inflammation and urinary tract infections.

### 2.7 Statistical Analysis

All reported values are presented with the mean standard deviation. For multiple comparisons, statistical analysis was performed using GraphPad Prism Software 5.04 and a one-way analysis of variance (ANOVA) followed by a post hoc Tukey’s test or the unpaired two-tailed Student’s *t* test technique. Statistical significance was defined as *p* values less than 0.05 (*), 0.01 (**), and 0.001 (***).

## 3 Results and Discussion

### 3.1 Synthesis and Characterization of Starting Materials

Gelatin–DOPA referred to as Gel–DOPA was synthesized by conjugating dopamine and phloretic acid to the gelatin backbone via a carbodiimide coupling using EDC and NHS as described above.^41^ This procedure yielded 0.042 mg/mL phenols in 1 mg/mL functionalized gelatin. The successful grafting of dopamine was further confirmed by the increased absorption at 220–240 nm due to catechol groups (Figure S1).^42^ Notably, no peaks appeared at 395 nm, which indicated that the latter groups were not oxidized during synthesis.^36^ In addition, we used the DPPH assay to further demonstrate the presence of DOPA, achieving concentration-dependent quenching of the free radical by the grafted phenolic groups (Figure S2).

**Figure 1:**
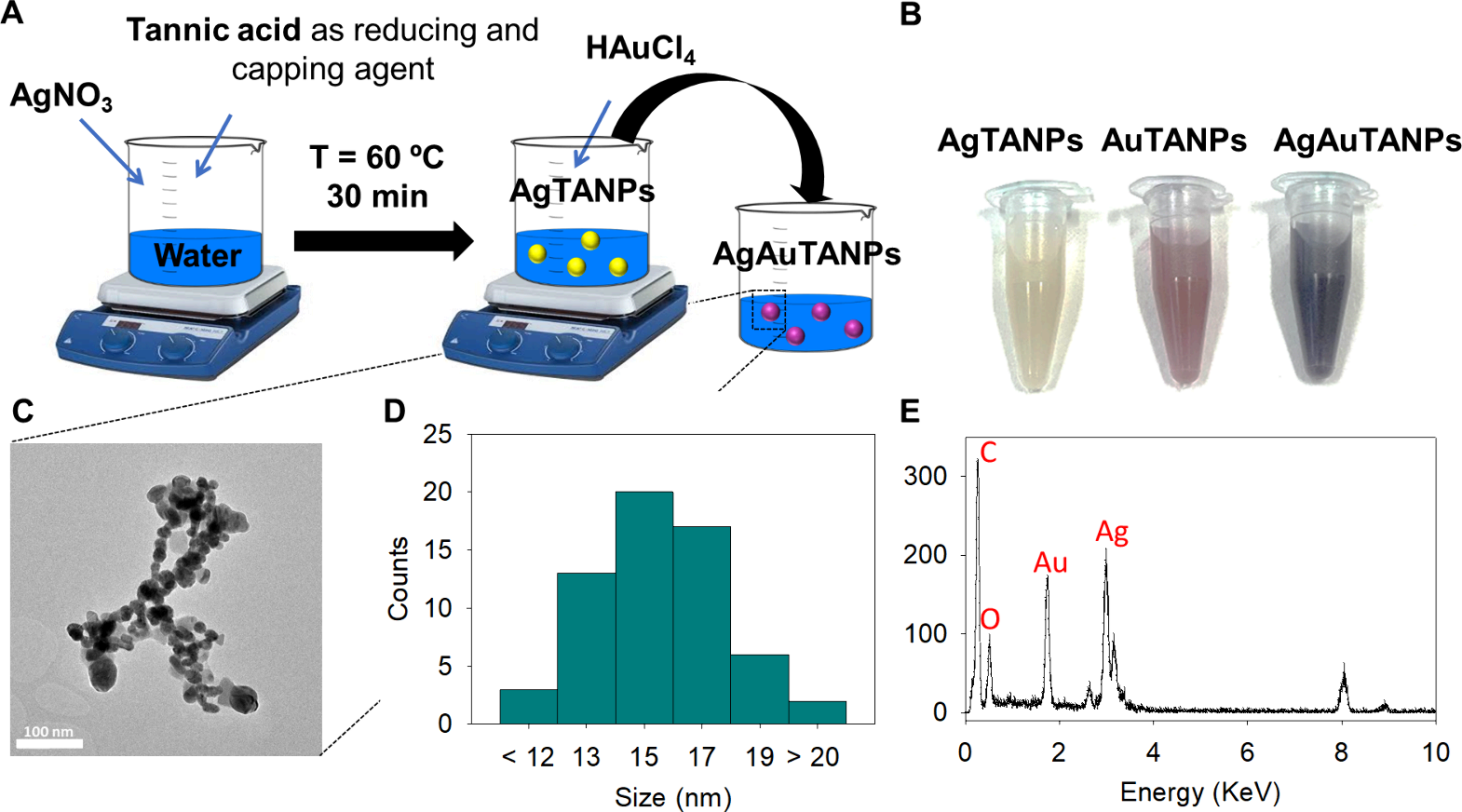
(A) Scheme of NP synthesis, (B) photographs of the NP suspensions, (C) representative TEM image of Ag/AuTANPs, (D) size distribution from TEM data, and (E) representative EDX profile (extracted from TEM) indicating bimetallic composition.

Metal–organic Ag/AuTANPs were obtained using tannic acid as both reducing and capping agent, according to the scheme in Figure 1A. The color changes of the silver nitrate and gold(III) chloride trihydrate solutions from colorless to gray-green and wine red, respectively, upon addition of tannic acid, were indicative of AgTANP and AuTANP formation. Furthermore, the addition of gold precursor to the dispersion of AgTANPs changed its color from gray-green to dark purple due to the spontaneous reduction of Au by the Ag core, generating bimetallic Ag/AuTANPs (Figure 1B).

The formation of NPs was monitored by using UV–vis spectroscopy (Figure S3). The appearance of a peak at 498 nm confirmed the presence of AgTANPs because Ag colloids exhibit maximum absorbance between 400 and 500 nm due to surface plasmon resonance (SPR).^43^ The same SPR effect resulted in a pronounced peak at about ∼540 nm for colloidal Au alone. Upon the deposition of Au on the AgTANPs, the absorption maximum shifted to 600 nm, indicating the combination of plasmon signals and ruling out the presence of individual AgTANPs and AuTANPs. In TEM studies, the generated bimetallic NPs exhibited a spherical morphology and a fairly similar size of 10–20 nm (Figure 1C,D), while in DLS, they appeared as up to 10-fold larger aggregates. Elemental mapping in TEM also confirmed both Au and Ag in each NP (Figure 1E). In addition, all NPs featured similar surface charges (−24 ± 1, – 21 ± 1, and −24 ± 1 mV for AgTANPs, AuTANPs, and Ag/AuTANPs, respectively) due to the same capping agent. During the Ag/AuTANPs synthesis process, phenols were likely partially oxidized but maintained 0.084 mg/mL of phenols in a 1 mg/mL Ag/AuTANPs solution, which confirmed the presence of tannic acid.

The bactericidal activity of the Ag/AuTANPs was determined by minimal inhibitory concentration (MIC) assays against *P. aeruginosa* and *S. aureus* and compared with the activity of AgTANPs and AuTANPs (Figure S4). Generally, the antimicrobial activity of metal NPs is related to their chemical composition and propensity for ion release.^44^ Due to the innocuous character of Au, AuTANPs did not exhibit a significant effect, while AgTANPs were more efficient in accordance with their known cytotoxicity. Importantly, the latter antimicrobial activity was potentiated in Ag/AuTANPs, lining with the notion that bimetallic combinations favor NP stability and promote the production of ROS.^45^

### 3.2 Physicochemical Characterization of the Sonochemical Coating

**Figure 2:**
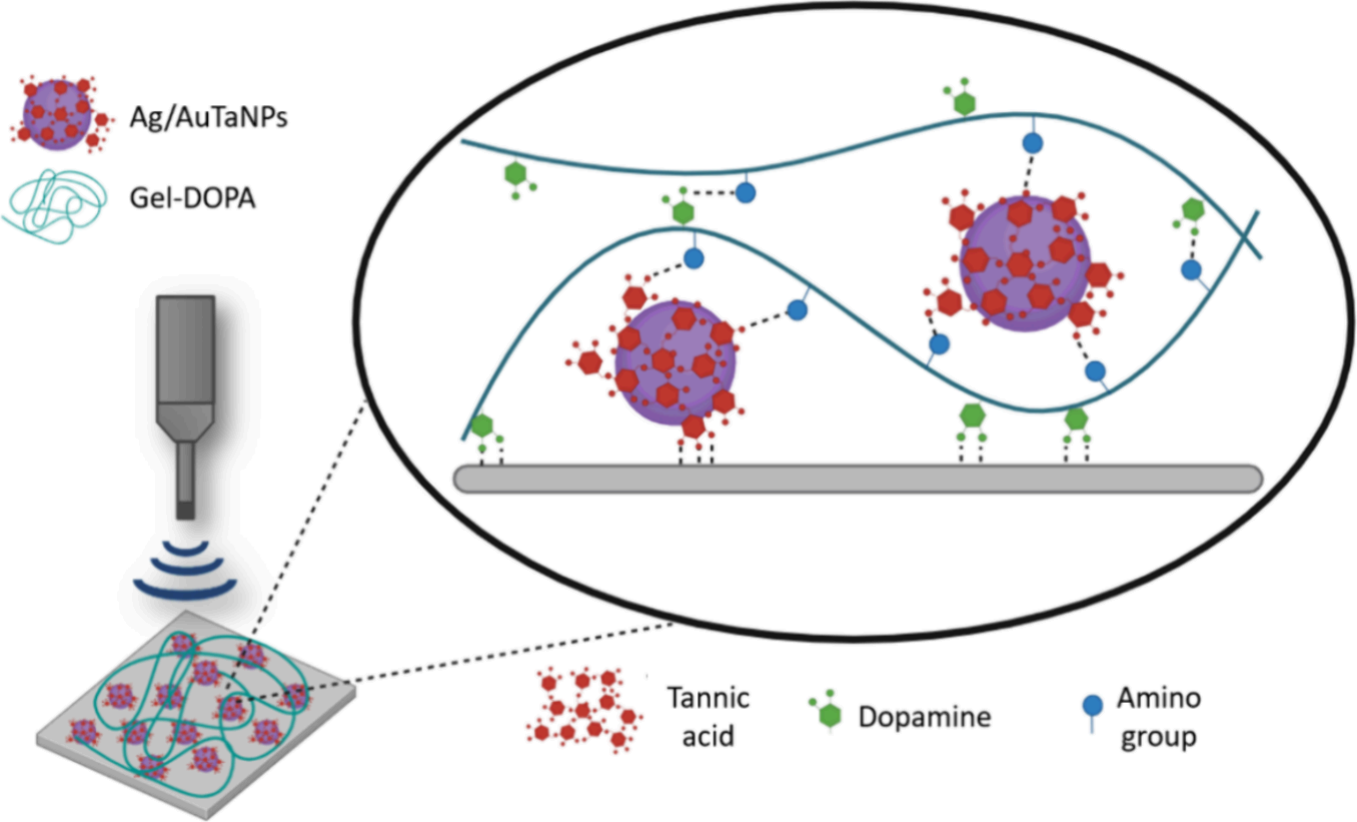
Scheme of the possible interactions in the sonochemical coating.

Antimicrobial Ag/AuTANPs and Gel–DOPA were simultaneously deposited on commercial Foley catheters by high-intensity ultrasound, avoiding the use of harsh reaction conditions or toxic compounds to enhance adhesion. During the sonochemical coating, NPs and Gel–DOPA were projected to the silicone substrate by cavitation, increasing the interaction between the modified biopolymer and the hydrophobic surface, while trapping in the bioadhesive the NPs^34^ (Figure 2).

**Figure 3:**
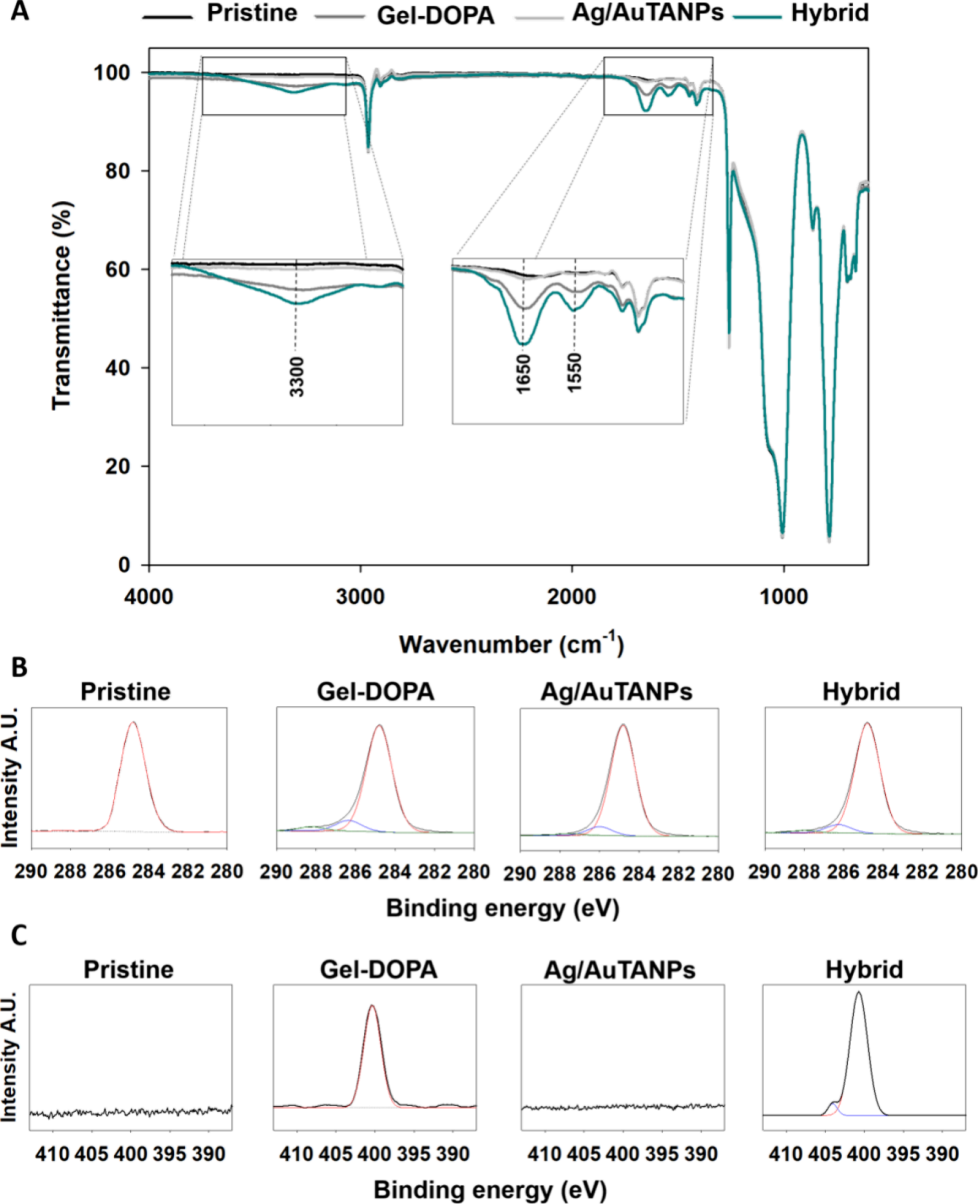
(A) FTIR spectra of different coatings. (B) High-resolution C 1s and (C) N 1s XPS spectra of the same samples (atomic composition in C 1s and N 1s, Tables S1 and S2).

The appearance of signals at approximately 1650 and 1550 cm^–1^ in the FTIR spectra, corresponding to stretching vibrations of C=O, and amide N–H vibrations, respectively, confirmed the presence of Gel–DOPA in the composite coating denoted as a hybrid. At the same time, stretching vibrations of N–H and O–H groups in the polymer conjugate resulted in wide bands at 3200–3400 cm^–1^ (Figure 3A). Interestingly, while coating with Ag/AuTANPs alone did not cause detectable changes compared to pristine silicone (likely due to low NP concentration on the surface), the NPs apparently increased the Gel–DOPA loading in the hybrid coating, as evidenced by the higher magnitude of the latter peaks.

Furthermore, XPS analysis revealed C–O/C–O-C (286 eV) and C=O/O–C–O/C–OH (288 eV) bonds in the C 1s spectrum arising from the functional groups in the gelatin–DOPA and Ag/AuTANPs (Figure 3B).^46,47^ In parallel, the amide peak in the N 1s spectrum of Gel–DOPA^48,49^ was augmented by another signal with higher energy (404 eV) in the hybrid sample (Figure 3C). It has been suggested that this interaction is initially driven by electrostatic attraction between the amino groups of gelatin and the NPs, ultimately leading to the formation of coordination complexes.^50,51^

**Figure 4:**
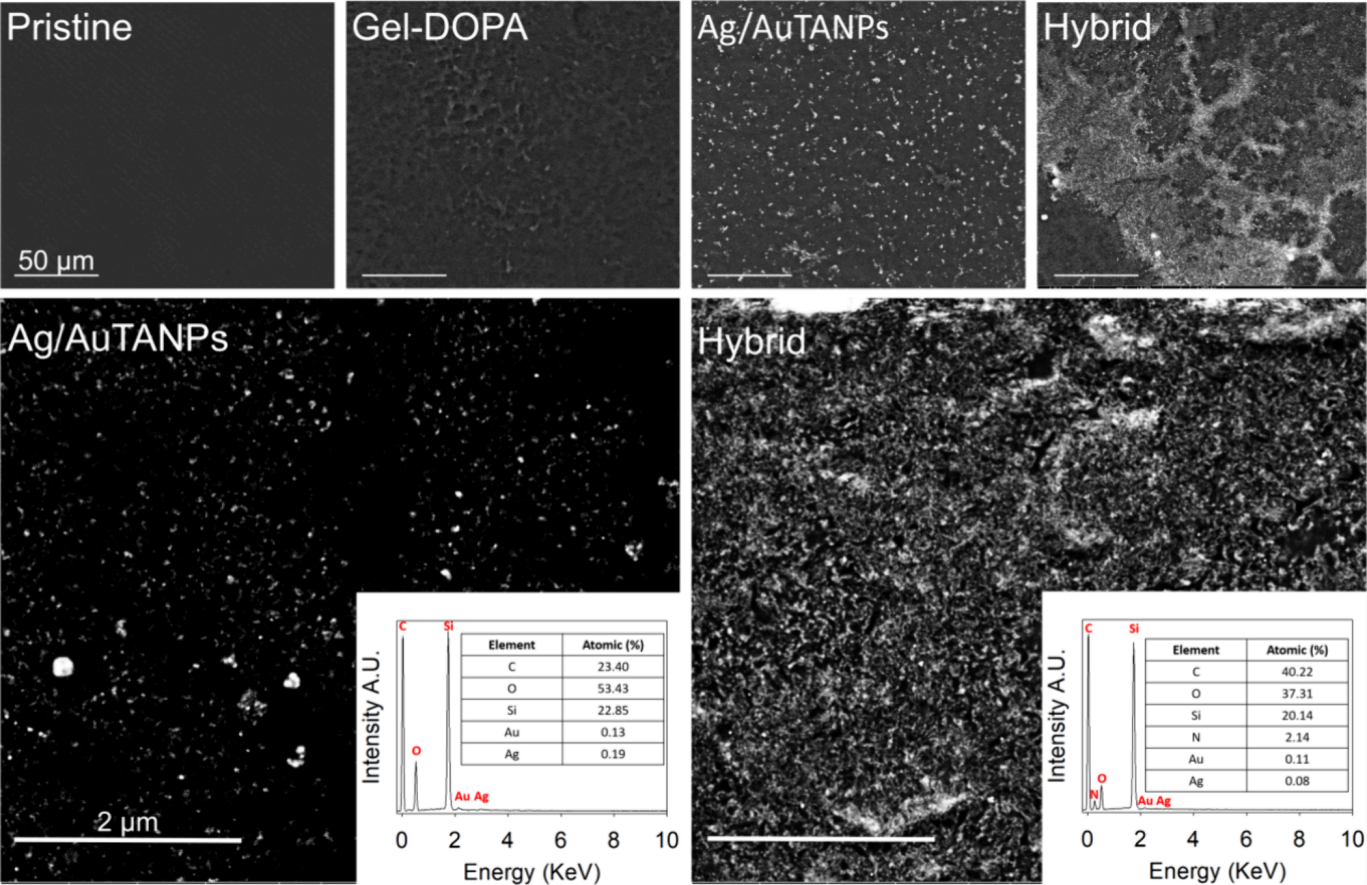
SEM micrographs of silicone surfaces after the sonochemical deposition of Ag/AuTANPs, Gel–DOPA, and Ag/AuTANPs_Gel–DOPA (hybrid).

The different coatings were also analyzed by SEM, which revealed a uniform distribution of dispersed bimetallic NPs on the silicone surface when deposited alone and apparently higher loading of NPs when deposited together with the modified gelatin (Figure 4).

**Figure 5:**
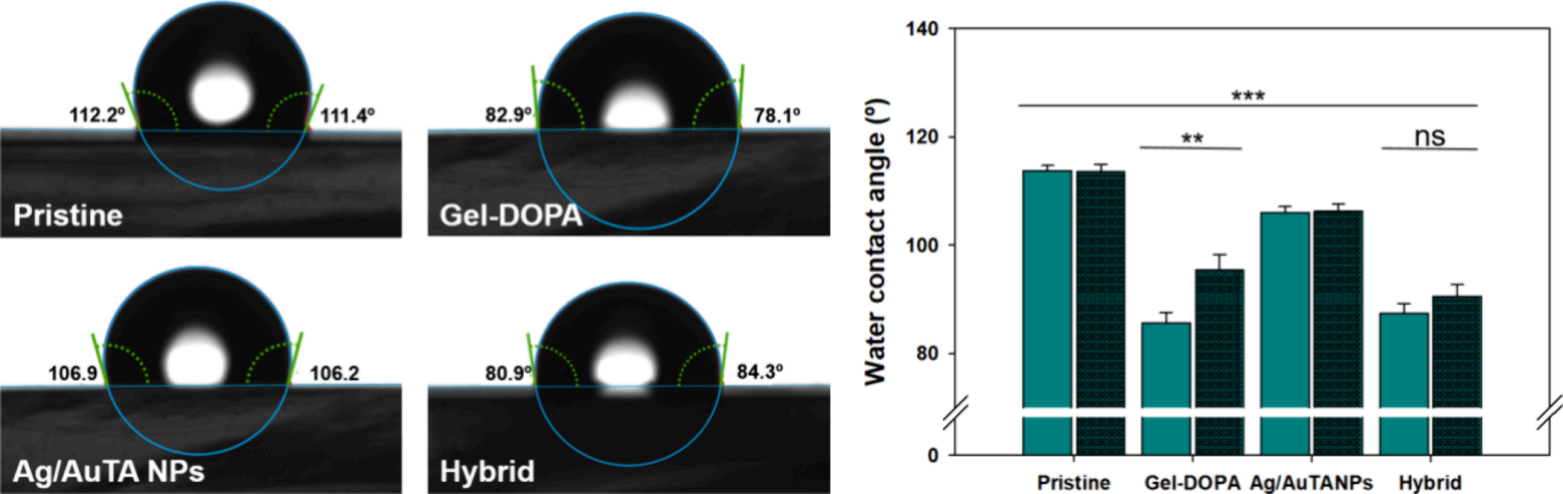
Water contact angle measurements of silicone surfaces with different coatings. Green bars represent measurements after functionalization, while dark green ones refer to measurements after 7 days of water incubation.

Functionally, successful deposition was confirmed through the reduction of the inherent hydrophobicity of the silicone. This effect was mainly attributed to the hydrophilic groups of the modified biopolymer as the major component of the composite coating because no significant difference was observed in comparison to Gel–DOPA alone (Figure 5). On the other side, the additional interactions with the Ag/AuTANPs in the hybrid coating increased its stability compared to Gel–DOPA (evidenced by contact angle measurements after 7 days of incubation), corroborating the structural role of the phenolic-shell NPs, and the stability of the coating.

**Figure 6:**
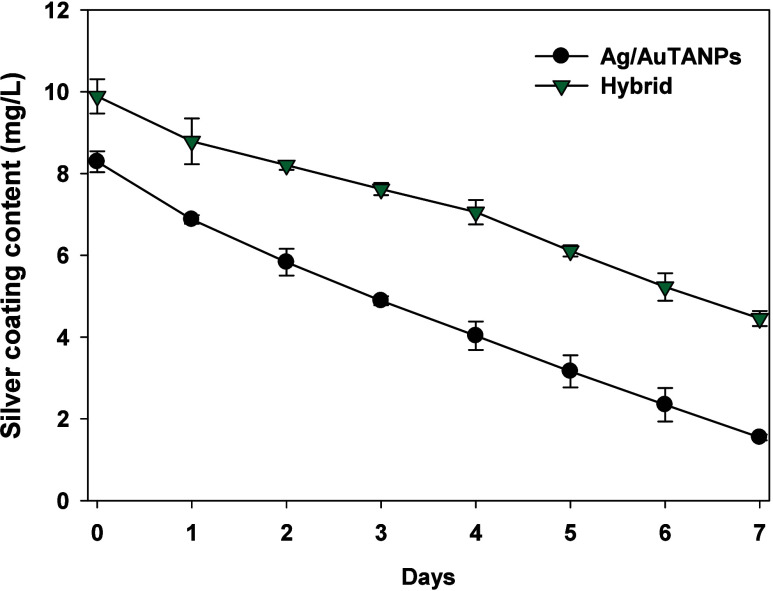
Silver release from 1 × 1 cm of silicone samples coated with Ag/AuTANPs alone and Ag/AuTANPs_Gel–DOPA (hybrid) after incubation in 2 mL of artificial urine during 7 days at 37 °C.

Next, ICP measurements indirectly quantified the amount of Ag/AuTANPs in the coating by assessing the release rate of Ag^+^, the latter being a functional determinant for durability and antibacterial efficacy. The “throwing-stone” mode of the ultrasonic process firmly embedded the Ag/AuTANPs onto the silicone surface,^52^ after which Ag^+^ was liberated in a fairly linear manner (Figure 6). Apparently, the tannic acid shell prevented a burst release, which could be expected for surface-adhered compounds. The resulting zero-order kinetics provide a practical material design variable where the intended dwell time (i.e., activity) can be matched to the initial loading. Furthermore, hybridization with Gel–DOPA increased the amount of Ag/AuTANPs on the surface in accordance with SEM observations (Figure 4) and slowed down the Ag^+^ release, maintaining 50% of residual Ag load after 7 days of incubation in artificial urine.

### 3.3 Antimicrobial and Antibiofilm Properties of the Coatings

**Figure 7:**
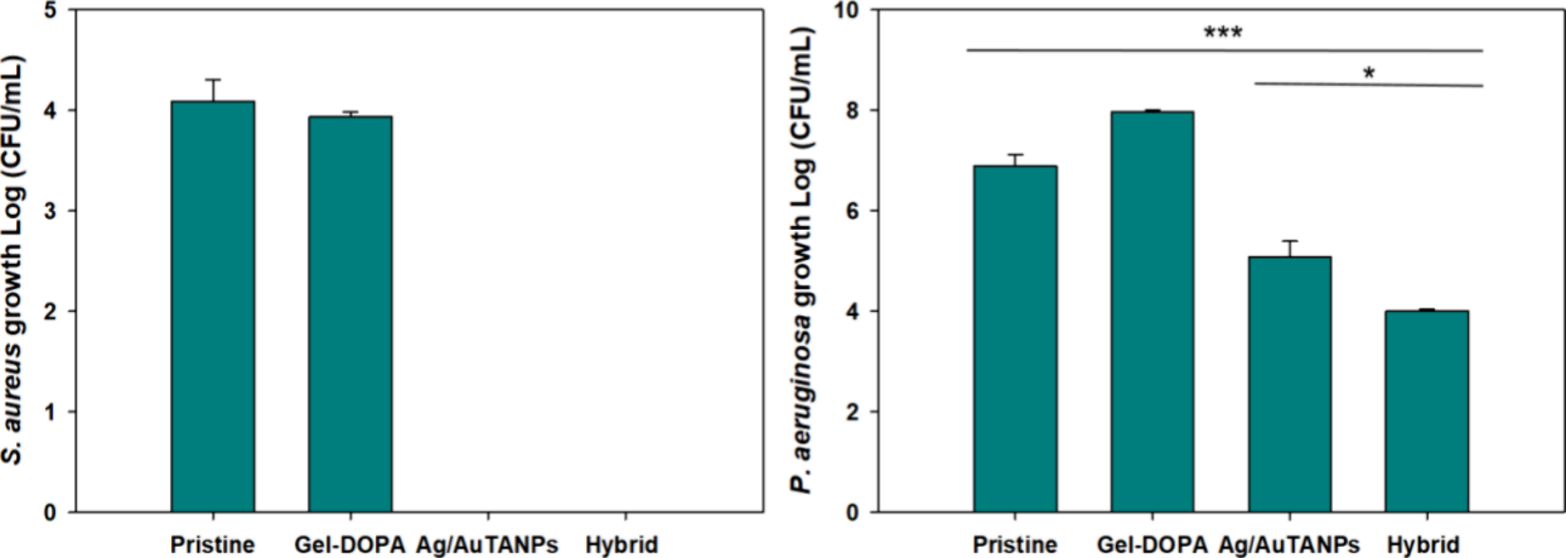
Antibacterial activity of the coated silicone materials toward *S. aureus* and *P. aeruginosa* after 24 h of incubation at 37 °C.

*S. aureus* and *P. aeruginosa* are two of the most frequent pathogens associated with CAUTI and were, therefore, used as models to assess the antimicrobial activity of the coatings. The latter were incubated with individual bacteria for 24 h, and as expected, no antimicrobial effect was observed with Gel–DOPA alone (Figure 7). On the other hand, samples coated with Ag/AuTANPs completely eradicated planktonic *S. aureus* and exhibited by 2–3 log reduction of *P. aeruginosa*, which can be linked to comparatively lower ROS production in the presence of the latter strain (Figure S5). Higher ROS levels in *S. aureus* compared to *P. aeruginosa* are likely due to the enhanced interaction of Ag/AuTANPs phenols with the bacterial peptidoglycan layer.^53^ Despite observing a higher release of Ag^+^ from the samples coated only with NPs, the hybrid coatings showed a stronger antibacterial effect, as revealed by the tests with *P. aeruginosa* (Figure 7). Thus, the potential shielding effect of Gel–DOPA (Figure 6) was apparently overshadowed by the increased NP loading (Figure 4), leading to significantly higher ROS production in the case of the hybrid coating (Figure S5A).

**Figure 8:**
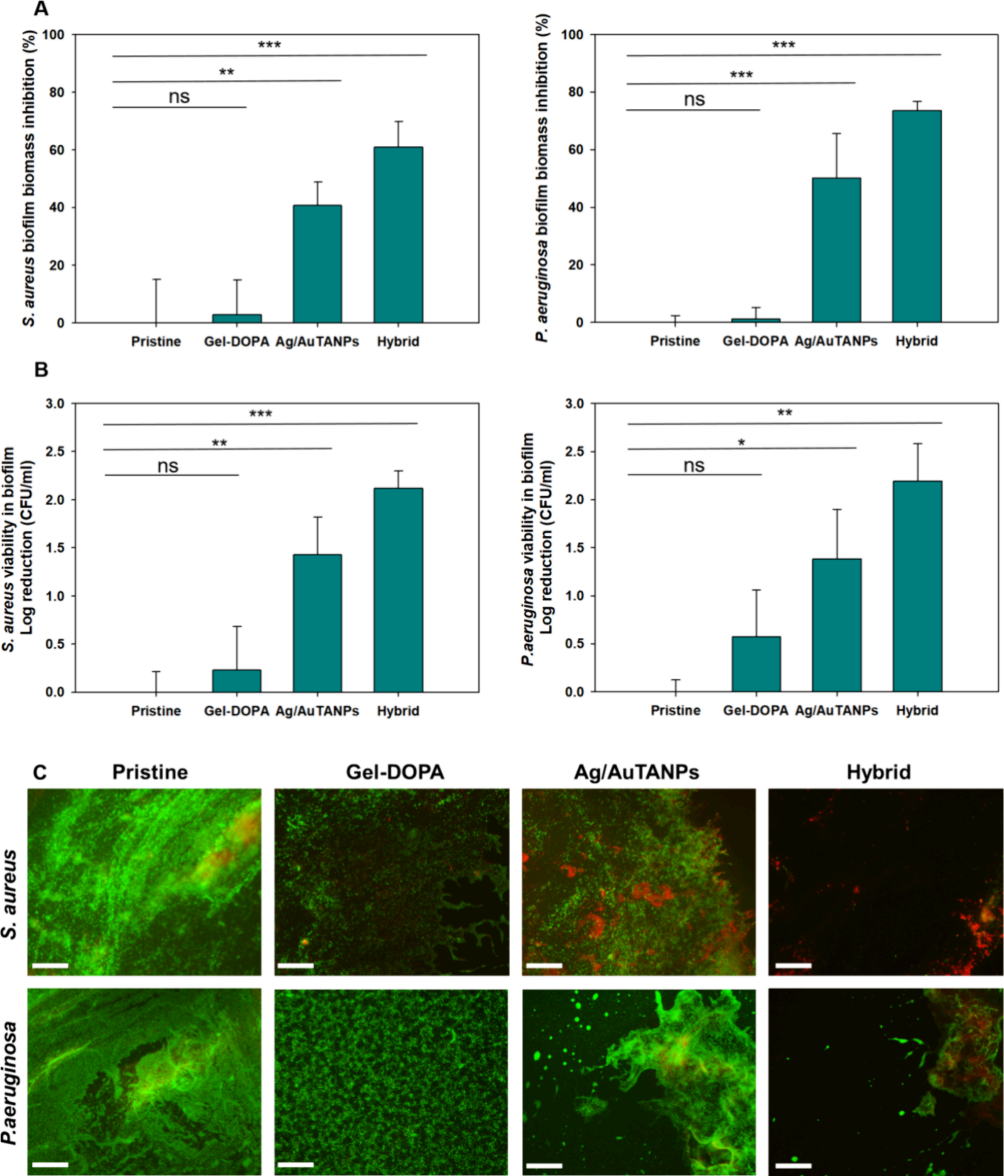
(A) Biofilm biomass inhibition and (B) cell viability of *S. aureus* and *P. aeruginosa* in biofilms cultured on different coatings under static conditions after 24 h of incubation. (C) Fluorescence microscopy images of live (green) and dead (red) *S. aureus* and *P. aeruginosa* cells on different coatings. The scale bar corresponds to 50 μm.

The prevention of biofilm formation was further assessed under static conditions by using crystal violet for quantification of the total biofilm biomass and bacterial viability assays. The hybrid coatings again surpassed those with Ag/AuTANPs alone, reducing the biofilm mass of *P. aeruginosa* and *S. aureus* by 60 and 70%, respectively (Figure 8A). Importantly, the bimetallic NPs can generate ROS not only in the presence of bacteria but also through extracellular reactions, as demonstrated in acellular experiments (Figure S5B). Thus, apart from direct effects on the bacterial redox cycle, ROS may disrupt quorum sensing or destroy exopolysaccharides and other matrix components, leading to overall reduced capacity to form biofilms.^54^ The trends between the different coatings (i.e., no effect of Gel–DOPA, and hybrids better performing than only NPs) was mirrored also in the data for bacterial viability, reaching about 2-log reduction (Figure 8B). Microscopic observations after staining with the Live/Dead kit were fully in line with the above quantitative outcomes (Figure 8C). Untreated silicone samples and those coated with Gel–DOPA exhibited well-established viable biofilms, while hybrids prevented biofilm formation more effectively than NPs alone did. Furthermore, the lower effect on *P. aeruginosa* was visually corroborated as well.

**Figure 9:**
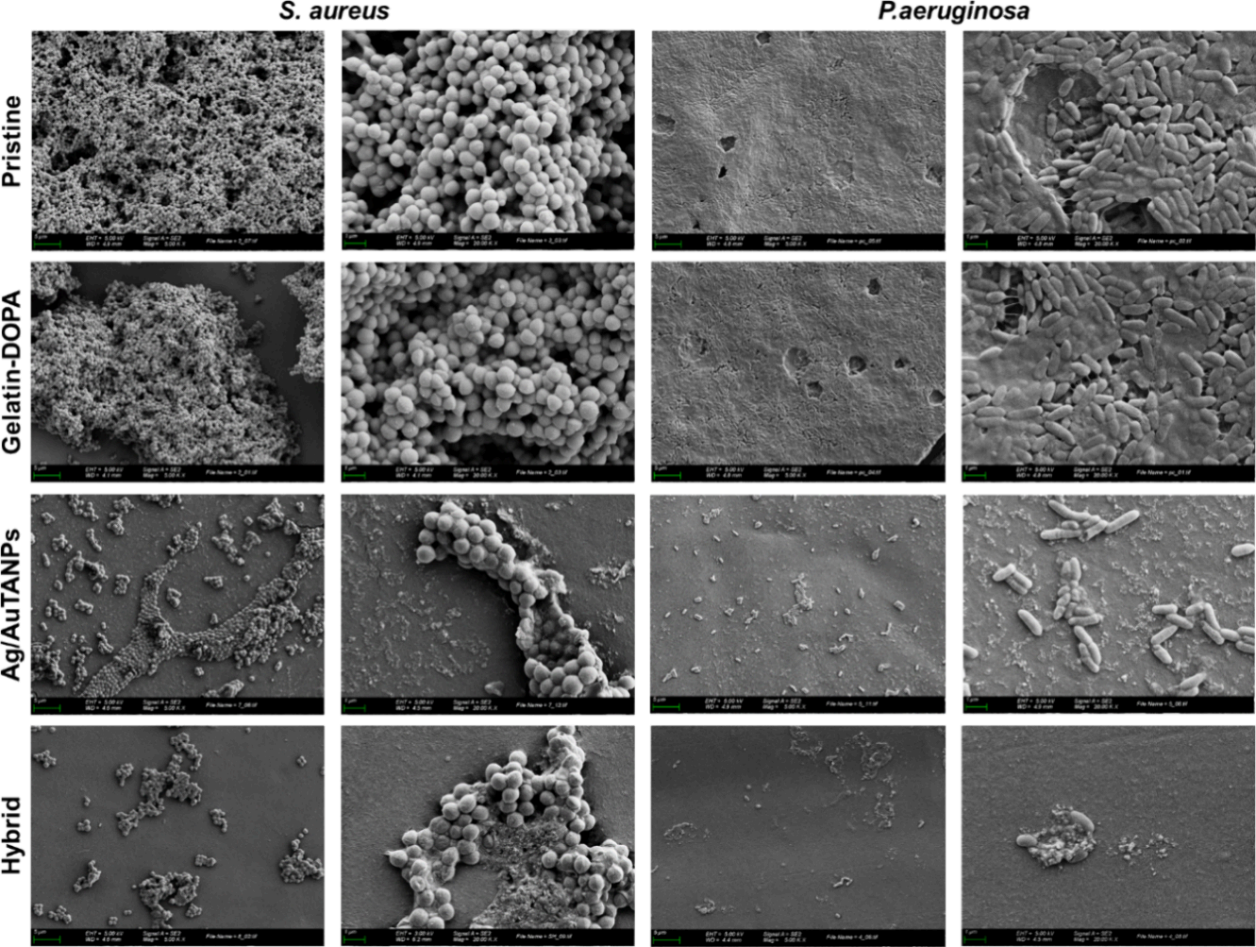
SEM images of *S. aureus* and *P. aeruginosa* biofilms grown on different coatings under static conditions for 24 h. The magnification of the SEM images is ×5000 and ×20,000.

SEM data further supported the observations above for the different coatings (Figure 9). Uniform biofilms were visible on untreated samples and those coated with Gel–DOPA, while in the samples containing Ag/AuTANPs, the biofilms were reduced and spread irregularly. In addition to the lesser adhesion to the surface, the latter samples evidenced morphological deformations and debris from burst cells.

**Figure 10:**
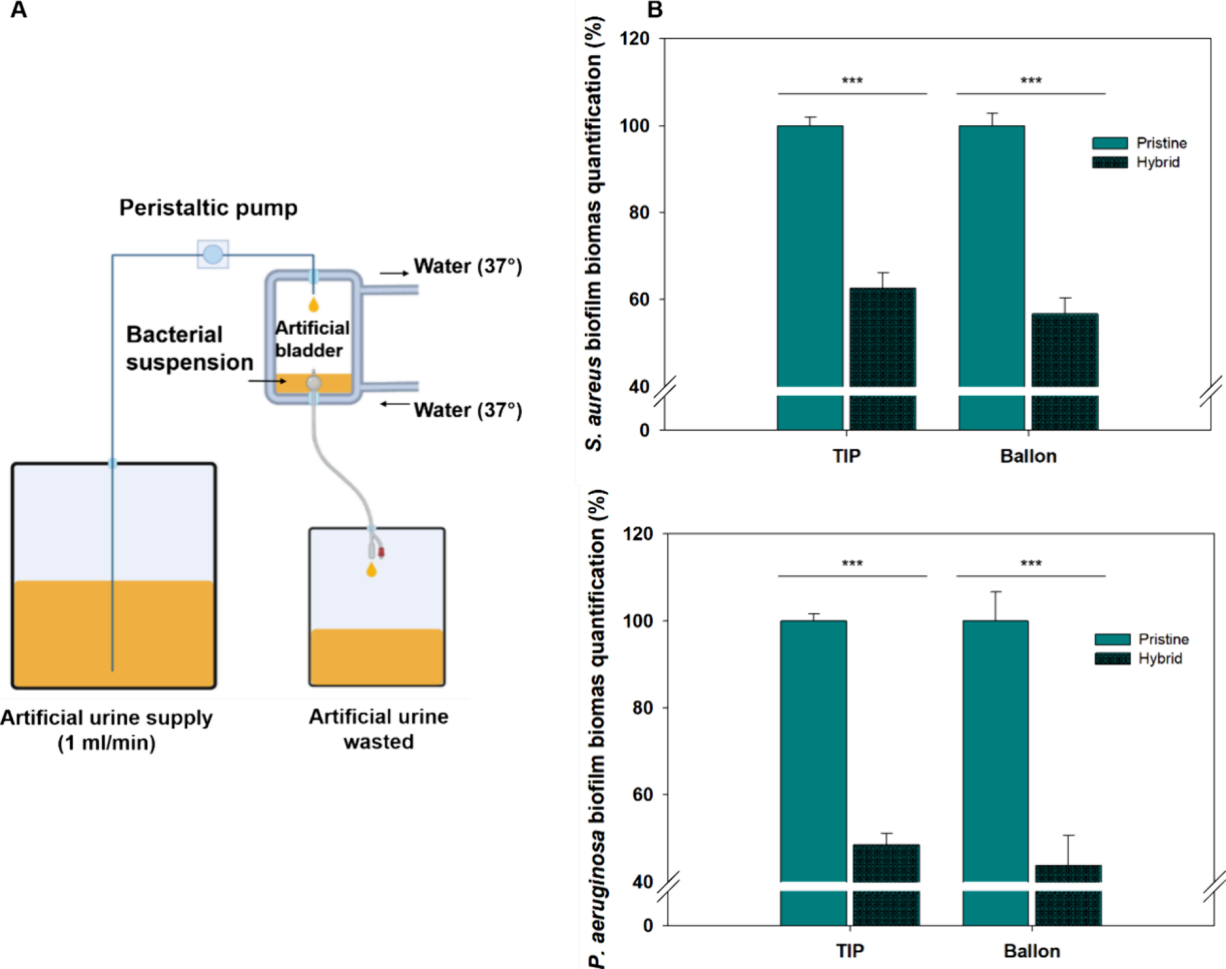
(A) Scheme of the artificial bladder setup for catheter testing. (B) Biofilm biomass quantification after dynamic bacterial exposure for 7 days at 37 °C in dynamic conditions. Green bars represent measurements after functionalization, while dark green ones refer to measurements after 7 days of dynamic incubation.

The durability of the hybrid coating was further tested via an in-house dynamic setup that simulated the urinary tract environment (Figure 10A). Pristine and coated catheters were inserted into said artificial bladders, which were inoculated and supplied with synthetic urine at a flow rate of 1 mL/min. These hydrodynamic conditions mimicked the free intraluminal flow in urinary catheters with a drainage bag, corresponding to the daily amount of urine produced by an adult person (0.8–2 L). After 7 days, the catheters were removed, cut, and stained with crystal violet to determine the biofilm biomass (Figure 10B). Indeed, the hybrid coating of Gel–DOPA and Ag/AuTANPs slowed the biofilm establishment even after prolonged testing. Furthermore, the antibiofilm activity fairly corresponded to the 24-h static tests. This indicated adequate coating stability under more realistic flow conditions owing to the interplay of the sonochemically applied metal NPs and the mussel-like bioadhesive.

### 3.4 Cytotoxicity

**Figure 11:**
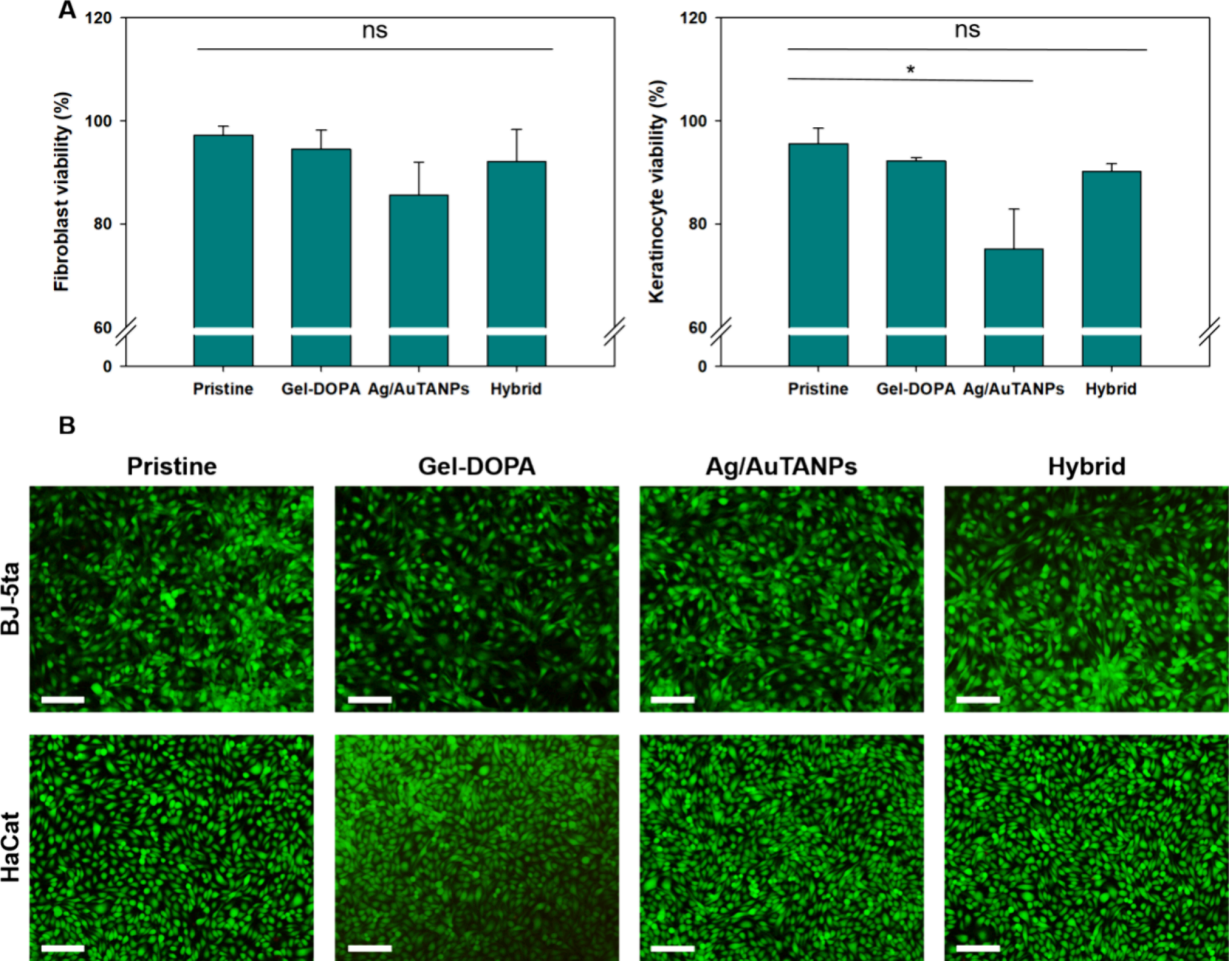
Viability of fibroblast and keratinocyte cell lines after exposure to different coatings. Micrographs of samples stained with (A) AlamarBlue and (B) Live/Dead kit assays. The green and red fluorescence signals are overlaid. The scale bar corresponds to 100 μm.

The coating biocompatibility is a critical parameter for the validation of indwelling medical devices and was explicitly addressed in the present case due to the potent but unspecific activity of Ag.^22^ To this end, fibroblasts and keratinocytes were placed in direct contact with different coatings for 24 h and then stained with AlamarBlue and Live/Dead kits. The latter microscopic method did not reveal morphological changes between the samples (Figure 9B), but quantitative data corroborated a certain cytotoxic effect of the bimetallic NP coating (Figure 11A). However, the Ag^+^ and NP release from the hybrid coating and the resulting ROS production were apparently modulated through the biocompatible matrix of Gel–DOPA and the capping tannic acid shell, resulting in no statistically significant changes in cell viability.

### 3.5 In Vivo Evaluation in a Rabbit Model

After promising antimicrobial and biocompatibility results, the hybrid coatings were finally validated in vivo and benchmarked against untreated silicone catheters. During the 7-day placement of the catheters, all animals exhibited a robust recovery process and maintained good overall health as evidenced by hematological analysis of several basic cell parameters (Tables S3 and S4). At the end of the experiment (Table S4), lower values for red blood cells and hemoglobin were measured in one rabbit from the group with coated catheters (experimental) and two from the control group compared to the initial values at the moment of catheterization, which were in the reference range. In addition, reactive thrombocytosis was observed in some cases. Blood biochemical analysis also indicated good overall health after 7 days of dwell time (Table S5) with patterns characteristic for stress and discomfort as observed in similar experimental designs.^55^ For instance, some rabbits exhibited higher creatine kinase and low creatinine because of minimized motility, while elevated glucose levels were characteristic for stress. Lower blood urea nitrogen and amylase in both groups were ascribed to possible malnutrition due to stress and the preventive usage of the collar. Microbiological analysis of urine taken directly from the bladder confirmed no bacteriuria at the beginning of the experiment. After 7 days of indwelling, *Enterococcus faecalis* was only occasionally detected in the experimental group (<10^3^ CFU/mL), while the control group with uncoated catheters revealed an abundance of *E. faecalis* and *S. aureus* (>10^5^ CFU/mL), which are pathogens, commonly found in CAUTI. Furthermore, white blood cells were absent in the urine of the experimental group, while controls showed increased levels, which is in fact not a typical infection response in rabbits and may indicate severe inflammation.^56^ The animals from the experimental group were attested to mild excess of urobilinogen and bilirubin and proteinuria (Table 1). Sediment smears were normal in all groups and typical for basic pH (Figure S6).

**Table 1:** Urinalysis of Individuals from Each Group (Rabbits 1, 2, and 3 with Uncoated Catheters and Rabbits 4, 5, and 6 with Coated Catheters) on the 7th Day of the Experiment^a^

**parameters**	rabbit 1	rabbit 2	rabbit 3	rabbit 4	rabbit 5	rabbit 6
WBC (cell/μL)	0	0	0	40	40	15
KET (mmol/L)	0	0	0	06	0.6	±0.5
NIT						
URO (μmol/L)				30	33	30
BIL (μmol/L)	0	0	0	8.4	86	8.6
GLU (mmol/L)	0	0	0	0	0	0
PRO (g/L)	0	0.3	0	≥3.5	≥3.5	0.3
SG	1.010	1.005	1.010	1.010	1.005	1.003
pH	8.0	8.0	8.0	8.0	8.0	8.0
BLD (cell/μL)	0	0	0	0	0	0
Vc (mmol/L)	0	0	0	0	0	0
MA (mg/L)	≤10	≥150	≤10	≥150	≥150	≥150
Ca (mmol/L)	7.5	7.5	7.5	7.5	7.5	7.5
CR (mmol/L)	≥26.4	≥26.4	≥26.4	≥26.4	≥26.4	≥26.4

a White blood cells (WBCs), ketone levels (KETs), nitrites (NITs), urobilinogen (URO), bilirubin (BIL), glucose (GLU), protein levels (PROs), urine specific gravity (SG), pH, blood cells (BLDs), urine vitamin C (Vc), microalbuminuria (Ma), calcium (Ca), creatinine (CR)

**Figure 12:**
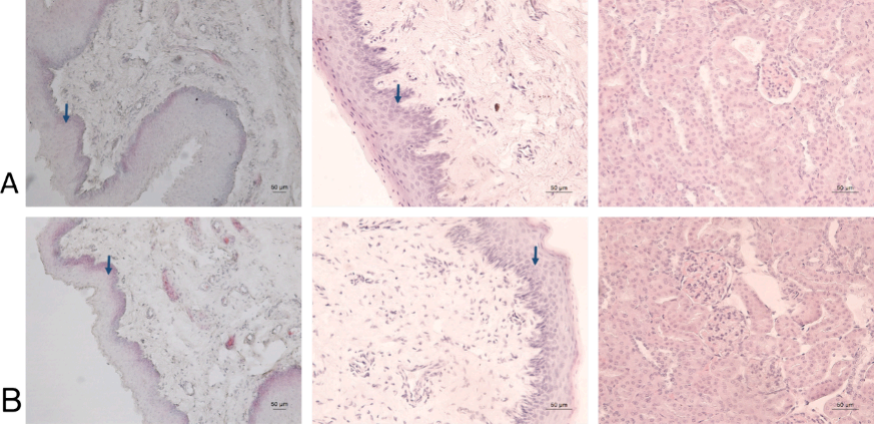
Histology of rabbit urethra: (A) an individual from the coated group; (B) an individual from the noncoated group. Left panel: prostatic part of the urethra; middle panel: urinary bladder, normal morphology of epithelium (urothelium) (blue arrows), and surrounding layers of loose connective and muscle tissue layers in both groups; right panel: renal corpuscles and convoluted tubules in kidney.

The histological structures of the rabbit urethrae from both groups did not show any deviations from the normal morphology after 7 days of catheterization (Figure 12). Along the course of the urethrae, well-developed urothelium with intact fibromuscular structures was observed. The bladders of all rabbits also revealed intact histology, despite the catheterization procedure. In both groups, renal cortex, medulla, convoluted tubules, and collecting ducts did not exhibit any signs of histopathological lesions, erosions, or inflammation.

The comprehensive examination of the clinical, histological, and microbiological data obtained from the rabbit models evidenced that the sonochemically coated catheters were biocompatible and effectively prevented CAUTI within 1 week of placement. In contrast, uncoated controls exhibited bacterial colonization and likely infection, also evidenced by elevated leukocyte esterase values in the range of 10–25 Leu/UL. Notably, the simultaneous presence of substantial quantities of *E. faecalis* and *S. aureus* (>10^5^ CFU/mL) in the control group corroborates the rapid infection during catheterization and the eminent risk of more severe inflammation, even in the absence of apparent histopathological changes during the test period.

## 4 Conclusions

In this study, we designed a mussel-inspired sonochemical coating, in which metal-phenolic NPs acted as both functional and structural elements to endow urinary catheters with preventive properties against CAUTI. The Gel–DOPA bioadhesive and the tannic acid shell of these NPs played a pivotal role for enhancing the coating stability and antimicrobial efficacy while reducing its cytotoxicity. In vitro studies showed that the combination of bimetallic NPs and a biopolymer effectively reduced biofilm formation, while measurements of ROS, DNA/protein release, and SEM observations provided further insights into their antimicrobial/antibiofilm mechanism. Thereby, the antimicrobial activity of the coatings correlated with the production of ROS, corroborating their importance for bacteria eradication. Importantly, the durability of the hybrid coating was challenged under realistic hydrodynamic conditions over a 1 week period, retaining its antimicrobial efficacy. Furthermore, there were no adverse effects on fibroblasts and keratinocytes cultured in direct contact with the coated samples. Finally, we substantiated the efficacy and safety of the functionalized catheters against CAUTI in an animal model, unequivocally validating superior performance in comparison to the pristine silicone catheters. Together with the simplicity of the waterborne sonochemical coating, these findings highlight the substantial practical potential of the Ag/AuTANPs_Gel–DOPA hybrid coating as a promising strategy for the antimicrobial functionalization of medical devices.

## Supporting Information Available

UV–vis spectra of coating precursors; antioxidant activity of functionalized gelatin; bacteriostatic activity of nanoparticles; ROS generation by different coatings; urine sediment smears; XPS spectra; blood parameters (PDF)
