# Supplementary material for: Mussel-Inspired Sonochemical Nanocomposite Coating on Catheters for Prevention of Urinary Infections
Source: ACS Appl Mater Interfaces. 2024 Jun 25;16(27):34656–68. doi: 10.1021/acsami.4c05713 (PMC11247429; doi:10.1021/acsami.4c05713)
Supplement: Supplementary file 1 — am4c05713_si_001.pdf [file am4c05713_si_001.pdf]

Supporting Information

Mussel-inspired sonochemical nanocomposite coating on catheters for prevention of urinary infections

Antonio Puertas-Segura,[1] Kristina Ivanova,[1] Aleksandra Ivanova,[1] Ivan Ivanov,[1] Katerina Todorova,[2] Petar Dimitrov,[2] Gianluca Ciardelli,[3] and Tzanko Tzanov[1]*

[1]Grup de Biotecnologia Molecular i Industrial, Department of Chemical Engineering, Universitat Politècnica de Catalunya, Rambla Sant Nebridi 22, 08222 Terrassa, Spain

[2]Institute of Experimental Morphology, Pathology and Anthropology with Museum, Bulgarian Academy of Sciences, Geo Milev, 1113 Sofia, Bulgaria

[3]Department of Mechanical and Aerospace Engineering, Politecnico di Torino, Corso Duca degli Abruzzi 24, 10129 Torino, Italy

*Author to whom correspondence should be addressed. E-mail: tzanko.tzanov@upc.edu

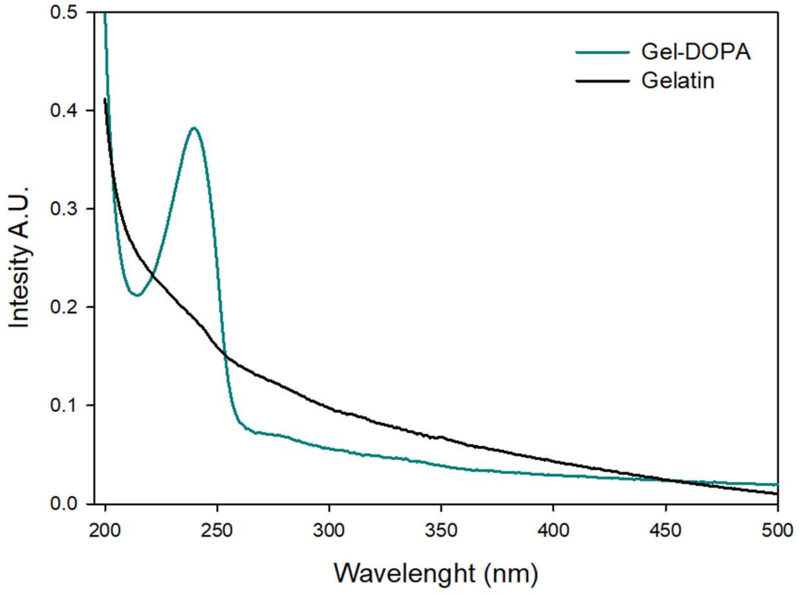

Figure S1. UV-Vis spectra of commercial gelatin and gel-DOPA

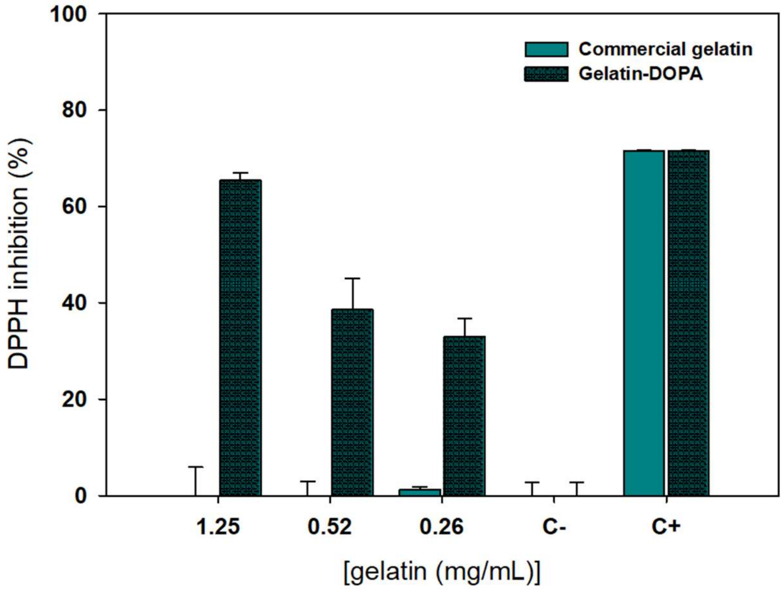

Figure S2. Antioxidant activity of gelatin and gelatin-DOPA. Negative control deionized water and positive control 1 mg/mL ascorbic acid solution.

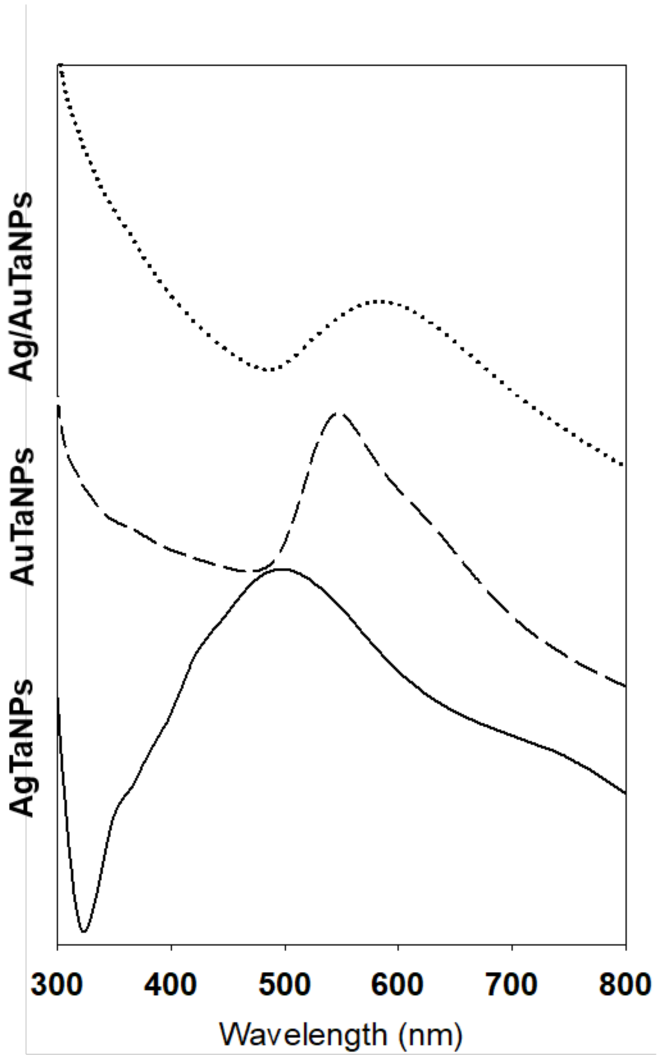

Figure S3. UV-Vis spectra of AgTANPs, AuTANPs, and Ag/AuTANPs suspensions.

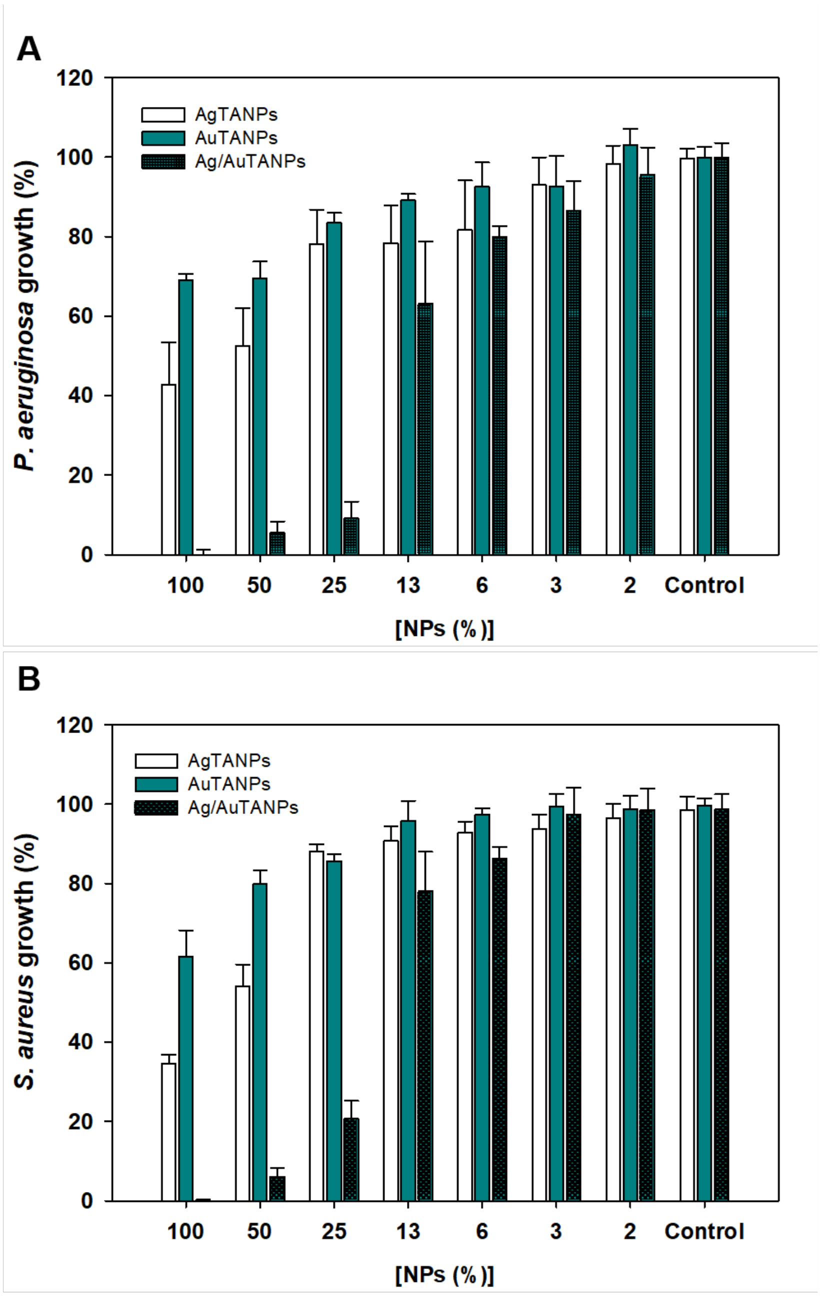

Figure S4. Minimal inhibitory concentration (MIC) of AgTANPs, AuTANPs, and Ag/AuTANPs against A) *P. aeruginosa* B) *S. aureus*.

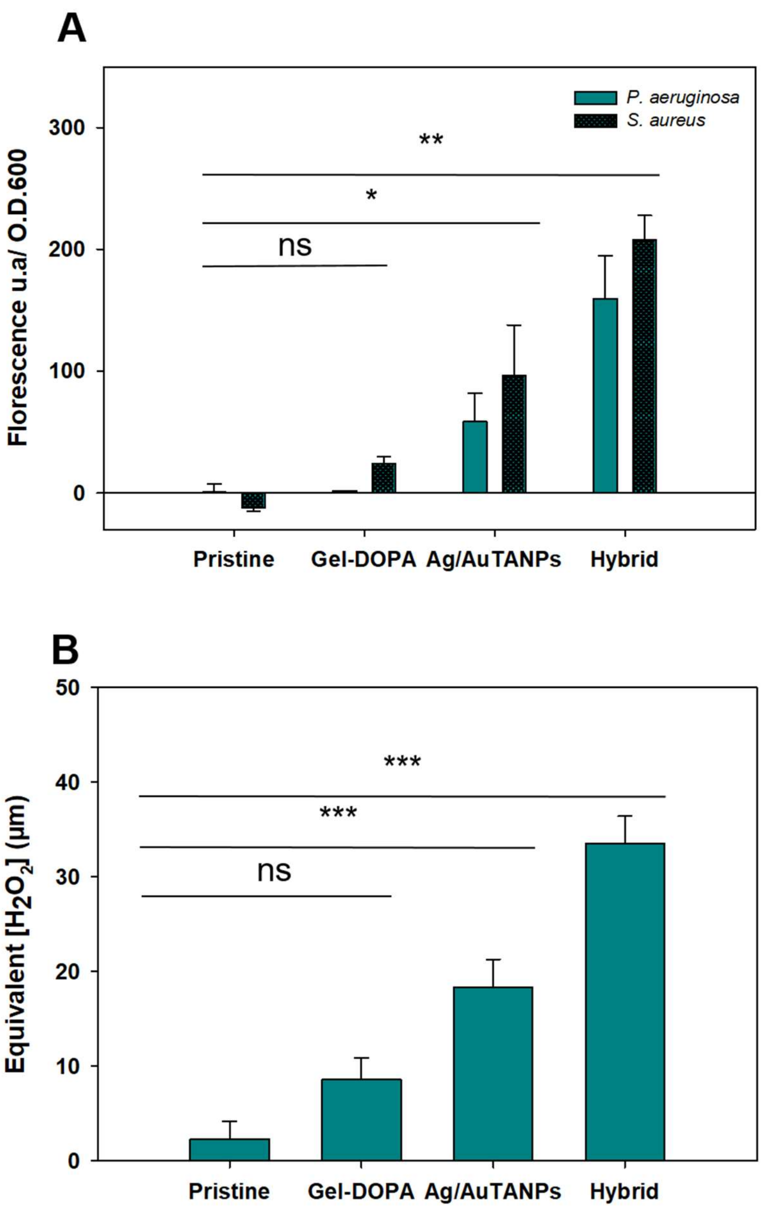

Figure S5. (A) ROS generation by bacteria in contact with differently treated silicones, assessed with the fluorescent probe DCFH-DA. (B) acellular ROS assay results.

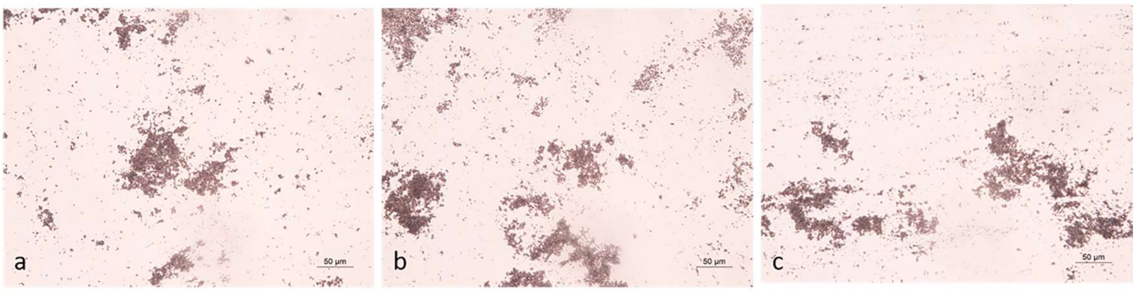

Figure S6. Sediment smears of urine from two male rabbits with a) and b) coated catheters, and c) uncoated ones. The clouds of Ca-containing microcrystals in all samples are normal findings in healthy rabbits.

Table S1. C 1s Energy binding of the deconvoluted high-resolution XPS spectra and functional group assignment.

C 1s	Atomic composition (%)		
Peak position (eV)	**284.9**	**286.4**	**287.5**
Bonds	**C-C/C-H**	**C-O/C-O-C**	**C=O/ O-C-O/ C-OH**
**Pristine**	100	0	0
**Gel-DOPA**	87.76	8.78	3.46
**Ag/AuTANPs**	91.12	7.26	1.62
**Hybrid**	90.99	6.90	2.11

Table S2. C 1s Energy binding of the deconvoluted high-resolution XPS spectra and functional group assignment.

N 1s	Atomic composition (%)	
Peak position (eV)	**400**	**404**
Bonds	**N-H/ R-NH/ C=O-NH**	**N-Metal/ RR´C=N-R**
**Pristine**	0	0
**Gel-DOPA**	100	0
**Ag/AuTANPs**	0	0
**Hybrid**	95.16	4.84

Table S3. Representative blood parameters of each group (rabbits 1, 2, and 3 with uncoated and rabbits 4, 5, and 6 with coated catheters) on the 1st day. White blood cell count (WBC), lymphocyte count (Lymph.), monocyte count (Mon.), granulocyte count (Gran.), red blood cell count (RBC), hemoglobin (HGB), platelet count (PLT).

**Parameters**	**Ref. Range**	**Rabbit 1**	**Rabbit 2**	**Rabbit 3**	**Rabbit 4**	**Rabbit 5**	**Rabbit 6**
WBC 10^9/L	5.2–13.5	10.0	10.5	10.5	13	12	11.6
Lymph. 10^9/L	3.2–9.0	3.7	3.9	4	5.5	7	6.5
Mon. 10^9/L	0.1–0.6	0.3	0.6	0.5	0.5	0.5	0.6
Gran. 10^9/L	2.0–7.5	6.0	6.0	6.0	7	4.5	4.5
RBC 10^{12}/L	5.0–7.6	5.9	5.9	5.9	5.5	5.5	5.5
HGB g/L	320–370	350	370	330	330	350	350
PLT 10^9/L	250–650	500	580	500	480	500	550

Table S4. Representative blood parameters from each group (rabbits 1, 2, and 3 with uncoated and rabbits 4, 5, and 6 with coated catheters) on the seventh day of the experiment. Legend as in Table S1.

Parameters	Ref. Range	Rabbit 1	Rabbit 2	Rabbit 3	Rabbit 4	Rabbit 5	Rabbit 6
WBC 10^9/L	5.2–13.5	10.5	10.5	12	13.4	14	13
Lymph. 10^9/L	3.2–9.0	4.1	4.5	6	7.9	7	7
Mon. 10^9/L	0.1–0.6	0.6	0.6	0.6	0.5	0.6	0.6
Gran. 10^9/L	2.0–7.5	5.8	5.4	5.4	5.0	6.4	5.4
RBC 10^{12}/L	5.0–7.6	↓4.8	5	5.4	↓ 4.9	↓ 4.8	5
HGB g/L	320–370	↓ 268	320	340	↓ 313	↓ 300	320
PLT 10^9/L	250–650	↑ 700	↑ 820	↑ 670	↑ 806	↑ 820	↑ 800

Table S5. Blood biochemical parameters from each group (rabbits 1, 2, and 3 with uncoated and rabbits 4, 5, and 6 with coated catheters) on the seventh day of the experiment. Albumin (ALB), total protein (TP), globulin (GLO), calcium (Ca), glucose (Glu), blood urea nitrogen (BUN), phosphorus (P), amylase (AMY), cholesterol (CHOL), alanine transaminase (ALT), total bilirubin (TBIL), alkaline phosphatase (ALP), creatinine (CRE), creatine kinase (CK).

Parameters	Ref. Range	Rabbit 1	Rabbit 2	Rabbit 3	Rabbit 4	Rabbit 5	Rabbit 6
ALB g/L	27–50	40.5	42.5	44.0	32.7	42.7	34
TP g/L	49–71	60.0	62.0	64.0	53.0	63.0	63.3
GLO g/L	15–33	19.5	19.5	20.0	20.3	20.3	29.3
Ca mmol/L	2.2–3.9	3.3	3.4	3.3	3.5	3.1	3.0
GLU mmol/L	5.5–8.2	↑ 12.0	↑ 13.0	↑ 10.0	↑ 10.0	↑ 14.0	↑ 13.0
BUN mmol/L	10.1–17.1	↓ 8.0	↓ 9.2	↓ 8.9	↓ 7.0	↓ 9.0	↓ 7.9
P mmol/L	1–2.2	1.0	1.5	1.2	1.2	1.5	1.5
AMY U/L	212–424	↓ 200	↓ 210	↓ 190	↓ 190	290	↓ 180
CHOL mmol/L	0.1–2	1.3	1.5	1.3	1.5	1.2	1.5
ALT U/L	27.4–72.2	60	56	70	68	66	68
TBIL μmol/L	2.6–17.1	3.2	3.7	8.3	15.3	17	15.6
ALP U/L	12–96	66	69	47	45	48	75
CRE μmol/L	74–1711	↓ 63	↓ 66	↓ 53	↓ 51	59	↓ 62
CK U/L	58.6–175	↑ 1250	↑ 1050	↑ 1350	↑ 1178	↑ 1100	↑ 890
